# Retracted: VGLL4 Protects against Oxidized-LDL-Induced Endothelial Cell Dysfunction and Inflammation by Activating Hippo-YAP/TEAD1 Signaling Pathway

**DOI:** 10.1155/2022/9851919

**Published:** 2022-04-28

**Authors:** Mediators of Inflammation

*Mediators of Inflammation* has retracted the article titled “VGLL4 Protects against Oxidized-LDL-Induced Endothelial Cell Dysfunction and Inflammation by Activating Hippo-YAP/TEAD1 Signaling Pathway” [1] due to concerns with figure duplication. It has been identified that the Western blot of VGLL4 in Figure 2(a) and Western blot of Caspase-3 in Figure 3(c) are identical when rotated 180 degrees.

The authors did not respond to our requests for clarification, and due to the above concerns, it is being retracted with the agreement of the editorial board.
